# Impact of exporter proteins and their engineering on the productivity of *Corynebacterium*

**DOI:** 10.1007/s00253-025-13479-1

**Published:** 2025-04-22

**Authors:** Keita Kinose, Keiko Shinoda, Hisashi Kawasaki

**Affiliations:** 1 Nagahama Institute for Biochemical Science, Oriental Yeast Co., Ltd., 50 Kano-Cho, Nagahama, Shiga 526 - 0804 Japan; 2 The Institute of Statistical Mathematics, Research Organization of Information and Systems, 10 - 3 Midori-Cho, Tachikawa, Tokyo 190 - 8562 Japan; 3 Graduate School of Agriculture and Life Sciences, The University of Tokyo, 1-1-1 Yayoi, Bunkyo-Ku, Tokyo 113 - 8657 Japan; 4 Research and Development Division, Kikkoman Corporation, 338 Noda, Noda City, Chiba 278 - 0037 Japan

**Keywords:** Synthetic biology, Metabolic engineering, Exporter engineering, Efflux improvement, MD simulation

## Abstract

**Abstract:**

Enhancing product efflux is crucial in improving fermentative bioproduction. Despite advancements in metabolic engineering guided by the design-build-test-learn cycle, membrane transport engineering of product efflux remains underdeveloped, limiting the efficient production of target chemicals. This review explores the historical findings on product efflux, regardless of passive or active transport, in fermentation engineering, focusing on *Corynebacterium* species, and highlights the potential of multidrug transporters as valuable screening sources for efflux improvement. Furthermore, the review emphasizes the importance of understanding the machinery of efflux transporters to optimize their functionality. Molecular dynamics simulations are a promising tool for exploring novel strategies to advance fermentation-related processes. These insights provide a framework for overcoming current challenges in membrane transport engineering of product efflux and improving industrial-scale bioproduction.

**Key points:**

*• Review of strategies to enhance product efflux in Corynebacterium species.*

*• Multidrug transporters are key tools for optimizing metabolite efflux.*

*• Efflux transporter mechanisms analyzed to improve microbial productivity.*

*• Molecular dynamics simulations employed for understanding transporter mechanisms.*

## Introduction

*Corynebacterium* species, commonly found in diverse environments such as soil and animal microbiota, include pathogenic and industrially significant species. Among the latter, *C. glutamicum* has gained significant attention from bioengineers since the discovery of its capacity for l-glutamate (Glu) production (Kinoshita et al. 1957; Udaka 1960). Over the years, multiple strains derived from this species have been developed to produce various amino acids, including l-lysine (Lys), l-threonine (Thr), l-serine (Ser), and Glu (Inui and Toyoda 2020).

In addition to *C. glutamicum*, *C. stationis* (formerly known as *C. ammoniagenes*) has emerged as an industrially important species due to its role in nucleotide fermentation. Engineered *C. stationis* strains can synthesize critical compounds for the food industry, such as inosine monophosphate (IMP), xanthine monophosphate (XMP), and guanine monophosphate (GMP) (Nara et al. 1967; Ledesma-Amaro et al. 2013). Additionally, riboflavin, a riboside compound as with nucleotide, can be produced by engineering this species (Koizumi et al. 2000; Pérez-García et al. 2021).

Traditionally, industrial strains have been developed through random mutagenesis followed by extensive screening focusing on analog resistance and auxotrophy to optimize metabolic performance. In addition to metabolic enzymes, mutations in transporters and channels have played a critical role in achieving high yields of desired compounds in *Corynebacterium* species. Product accumulation in the culture supernatant facilitates higher titers and simplifies downstream purification compared with intracellular extraction. Mutagenesis-based screening for secretory phenotypes of amino acids and nucleotides made the process practical. The later researches revealed critical mechanisms underlying secretion. For instance, Glu export in *C. glutamicum* is improved by mutations in the mechanosensitive channel NCgl1221 (Nakamura et al. 2007), while enhanced transport of IMP from *C. stationis* cells is facilitated by the active transporter Cs0286 (Kinose et al. 2024).

*Corynebacterium* species have continued to attract significant attention since the advent of biotechnology, owing to their exceptional metabolic activity and suitability as chassis organisms in synthetic biology. Advances in strain engineering have enabled the production of a wide range of bio-based platform chemicals, polymer substrates, and bioactive compounds. Examples include lactate, succinate (Tsuge et al. 2015; Das et al. 2024), shikimate (Kogure et al. 2016), 1,5-diaminopentane (cadaverine, Mimitsuka et al. 2007; Leßmeier et al. 2015), and 5-aminovaleric acid (Shin et al. 2016; Jorge et al. 2017).

In modern strain development, the DBTL cycle approach based on synthetic biology has been employed instead of random screening methods. This systemic approach involves a high-throughput and automated cycle of *designing* enzymatic reaction pathways, *building* expression systems, *testing* them under scalable fermentation conditions, and *learning* processes to refine metabolic models based on experimental outcomes (Petzold et al. 2015).

Although nearly a decade has passed since the DBTL paradigm was introduced, the methodology for enhancing membrane transport remains less advanced than metabolic modification. This review aims to explore historical findings on efflux enhancement of valuable chemicals in *Corynebacterium* species and propose systematic strategies for improving efflux mechanisms to address the lack of systematization in this critical area of fermentation engineering.

## Exporter proteins involved in bioproduction using *Corynebacterium* species

This section outlines representative exporter proteins that facilitate the export of valuable low-molecular-weight compounds, such as amino acids, in *Corynebacterium* species.

### Glutamate

Glu is primarily used as a seasoning due to its umami taste and as an intermediate in pharmaceutical synthesis. The estimated global demand for Glu, primarily as its monosodium salt, is approximately 3.56 million tons annually (2023). *C. glutamicum* was discovered in 1957 as a glutamate-producing microorganism (Kinoshita et al. 1957; Udaka 1960), marking a significant milestone in microbial biomanufacturing, enabling the industrial production of amino acids essential for protein synthesis.

Glu biosynthesized within the cell is exported through mechanosensitive channels (Msc) that respond to membrane tension (Nakamura et al. 2007; Hashimoto et al. 2010, 2012; Börngen et al. 2010). *C. glutamicum* expresses two types of Msc: the MscS-type, which opens at lower membrane tension with lower conductance, and the MscL-type, which opens at higher membrane tension with higher conductance. Among these, MscCG (NCgl1221), an MscS-type channel, is primarily responsible for Glu export during fermentation. Some strains possess only MscCG, while others contain a second MscS-type channel MscCG2 (Nottebrock et al. 2003; Wang et al. 2018; Nakayama et al. 2018). However, MscCG is dominant in Glu export, even in strains possessing both types. In strains with disrupted genes for both MscCG and MscCG2, extracellular Glu accumulation is negligible (Wang et al. 2018).

In addition to Glu, MscCG exports other low-molecular-weight compounds, such as aspartate and phenylalanine (Hashimoto et al. 2010, 2012). MscCG comprises approximately 530 amino acid residues, nearly double the size of the well-studied MscS from *Escherichia coli* (286 residues). Its N-terminal amino acid sequence shares similarities with *E*. *coli* MscS, whereas the C-terminal sequence is unique to *Corynebacterium*. In contrast, MscCG2, with approximately 330 amino acid residues, is closer in size and structure to *E. coli* MscS (286 amino acid residues) and features three transmembrane domains.

MscCG has a lower activation threshold for membrane tension than *E*. *coli* MscS and MscCG2 (membrane tension for half activation (mN/m): MscCG ~ 5.5 mN/m; MscCG2 ~ 12 mN/m, *E*. *coli* MscS ~ 12 mN/m), in which the C-terminal region including the fourth transmembrane domain is involved (Nakayama et al. 2016, 2018). The fact that MscCG opens at relatively low membrane tension is considered the basis for its function as the main export protein of Glu during Glu fermentation. In addition, MscCG does not close easily, even when membrane tension is released under physiological membrane potential, and this characteristic contributes to Glu fermentation (Nakayama et al. 2018).

Under normal culture conditions, mechanosensitive channels are not activated. In wild-type strains, external triggers such as penicillin are required to induce Glu production. In such scenarios, amplifying MscCG enhances extracellular Glu accumulation. Several gain-of-function mutations have been identified in MscCG, enabling it to activate at lower membrane tensions (Nakayama et al. 2012). These mutations allow Glu accumulation without external induction (Nakamura et al. 2007). Similarly, gain-of-function mutations in MscCG2 enable extracellular Glu accumulation under non-inducing conditions (Wang et al. 2018). Although MscCG2 requires higher membrane tension for activation, it exhibits higher transport capacity than MscCG (conductance: MscCG2 ~ 1.0 nS, MscCG ~ 0.34 nS), making it a promising target for engineering as an export protein (Nakayama et al. 2018).

Functionally improved variants of MscCG2 have been developed using error-prone PCR. These variants demonstrate enhanced Glu export capabilities due to structural modifications, such as enlarging the intracellular Glu portal or widening the narrowest part of the Glu passage (Nie et al. 2021). Recent advancements in the structural engineering of MscCG2 include enlarging the intracellular Glu portal and reducing its negative charge to mitigate electrostatic repulsion between Glu and the channel (Nie et al. 2024).

The high substrate tolerance of Msc, such as MscCG, highlights their potential as versatile export proteins in bioproduction (Kawasaki and Martinac 2020). In fact, it has been reported that expression of the MscCG genes with gain-of-function mutation in *E*. *coli* in which phenylalanine (Phe) biosynthesis was enhanced significantly improves Phe production (Hashimoto et al. 2012).

### Lysine

Lysine (Lys) is widely used as a feed additive and in medical amino acid infusions, with a global demand estimated at 2.6 million tons annually as hydrochloride salt (2018). Currently, *C*. *glutamicum* and *E*. *coli* are the primary microorganisms employed for Lys production.

In *C*. *glutamicum*, intracellularly synthesized Lys is exported by LysE, a member of the LysE protein family (Vrljic et al. 1996). The discovery of LysE marked the beginning of molecular biological analyses of amino acid export proteins in microorganisms. Since LysE-knockout strains fail to accumulate Lys extracellularly, LysE is essential for Lys production (Vrljic et al. 1996). Amplifying the LysE gene in the wild-type background increased extracellular Lys accumulation approximately fivefold (Vrljic et al. 1996).

In contrast, a Lys-producing strain engineered through the incorporation of five beneficial mutations into the wild-type strain—mutations originally identified through traditional random mutagenesis and used in industrial production—achieved sufficient industrial performance without artificial amplification or amino acid substitution of the LysE gene (Ikeda et al. 2006).

This finding suggests that the current level of LysE gene expression, regulated endogenously by the positive regulator LysG, is sufficient for Lys production. LysE also facilitates the export of d-Lys, arginine, and l-lysine (Stäbler et al. 2011; Bellmann et al. 2001).

Recently, a novel Lys exporter, MglE, belonging to the EamA superfamily, was identified through metagenomic screening of bovine intestinal microbiota. When the MgIE gene was introduced into a Lys-producing strain of *C*. *glutamicum*, which initially accumulated 27.1 g/L of Lys, extracellular Lys accumulation improved to 29.7 g/L (Malla et al. 2022).

### Threonine

Threonine (Thr) is primarily used as a feed additive, with an estimated global demand of approximately 700,000 tons/year (2018). Thr export to the extracellular space is mediated by ThrE, a member of the ThrE protein family (Simic et al. 2001). ThrE also facilitates the export of l-serine (Simic et al. 2001). In a strain accumulating 48.6 mM Thr, ThrE gene amplification using a plasmid increased Thr accumulation to 67.6 mM (Simic et al. 2002). Additionally, the heterologous expression of Thr export proteins from *E*. *coli*, such as *rhtA* or *rhtC*, significantly enhanced extracellular Thr accumulation in *C. glutamicum* (Diesveld et al. 2008). These results highlight the potential of heterologous exporters to improve export efficiency. If another organism has an exporter of the target chemical that is not inherently possessed by the microorganism that has enhanced biosynthesis of the target chemical, it is thought that heterologous expression of that exporter would be effective. Since there are still many exporters for which the substrates have not been studied in detail, in order to achieve the above, detailed research will be necessary in the future to clarify substrates for many exporters.

### Branched-chain amino acids and methionine

l-isoleucine (Ile), l-leucine, l-valine, and l-methionine are exported via BrnEF, a transporter comprising BrnE and BrnF, which belongs to the branched-chain amino acid exporter (LIV-E) family (Kennerknecht et al. 2002; Trötschel et al. 2005). The transcription of BrnEF is upregulated by a leucine-responsive protein (Lrp) in response to increased intracellular substrate concentrations (Vogt et al. 2014). Although BrnEF amplification is not strictly required for producing strains, co-expression of BrnEF and Lrp in an Ile-producing strain of *C*. *glutamicum—*developed through random mutagenesis—enhanced the l-isoleucine yield per consumed sugar by 1.6-fold. This modification also improved extracellular accumulation and production rates (Yin et al. 2013).

### Serine

SerE, a homolog of the EamA family export proteins in *E*. *coli*, has been identified as the primary exporter of Serine (Ser) in *C*. *glutamicum* (Zhang et al. 2020). SerE facilitates the export of both Thr and Ser. In addition to SerE, NCgl0254 and NCgl0255 contribute to Ser export, although SerE plays the predominant role (Gao et al. 2024). In an engineered Ser-producing strain accumulating approximately 20 g/L of Ser extracellularly, the disruption of the SerE gene reduced Ser accumulation to 11.3 g/L (Zhang et al. 2020). Conversely, amplifying the SerE gene in a Ser-producing strain improved extracellular Ser accumulation by approximately 10% (Zhang et al. 2020). Furthermore, overexpressing all four Ser exporters (SerE, NCgl0254, NCgl0255, and ThrE) in the A36 strain, which originally accumulated 31 g/L of Ser, increased extracellular Ser accumulation to 41.1 g/L. (Gao et al. 2024).

### Arginine

Arginine (Arg) is mainly exported by LysE, while CgmA (Cg2893) also has an ability to export Arg (Bellmann et al. 2001; Lubitz et al. 2016).

### Succinate

Succinate is widely used as a seasoning and a component in biodegradable polymers. Various microorganisms produce succinate through a reductive reaction in the TCA cycle. Under oxygen-limited conditions, the facultative anaerobe *C*. *glutamicum* synthesizes succinate from sugars via a reductive TCA cycle reaction. Succinate export is mediated by SucE, a member of the AAEx family (Fukui et al. 2011; Fuhn et al. 2011), as well as CgynfM, which also excretes other dicarboxylates (Fukui et al. 2019).

In a SucE-knockout strain, intracellular succinate levels increase, while extracellular succinate decreases by approximately 30% (Fukui et al. 2011). Amplification of the SucE gene in a succinate-producing strain with disrupted lactate dehydrogenase led to an increase in succinate accumulation from 174 to 274 mM (Fukui et al. 2011), while further strain engineering also achieved a succinate concentration of 1.13 M without SucE amplification (Litsanov et al. 2012). These results suggest that the promotion of succinate export by amplifying SucE gene contributes to the improvement of production, and that on the other hand, succinate export does not become a bottleneck at least in the enhancement of biosynthesis to some extent because SucE is an inducible protein.

### Diaminopentane

1,5-Diaminopentane, a valuable raw material for polyamides, is synthesized via lysine decarboxylation.

A 1,5-diaminopentane-producing strain was developed through heterologous expression of Lys decarboxylase in a Lys-producing strain, and its export is mediated by CgmA (Cg2893), a member of the major facilitator superfamily (MFS) (Kind et al. 2011). Disruption of CgmA reduced extracellular accumulation of 1,5-diaminopentane by 90%, whereas increased expression enhanced its production (Kind et al. 2011). Additionally, co-expressing a lysine/cadaverine antiporter from *E*. *coli* with Lys decarboxylase further improved accumulation in *C*. *glutamicum* (Li et al. 2014).

### Purine base analogs

Overexpression of the CepA gene in *C*. *glutamicum*, a homolog of NepI that exports purine nucleosides in *E*. *coli*, improved resistance to purine base analogs (6-mercaptopurine and 6-mercaptoguanine) but had no effect on resistance to purine nucleoside analogs. Interestingly, high CepA expression also enhanced resistance to antibiotics such as nalidixic acid (Sim et al. 2014).

## Multidrug efflux transporters in bioproduction processes

Recent advances in microbial fermentation have enabled not only for producing primary metabolites with *Corynebacterium* species but also larger and more complex secondary metabolites with metabolically engineered microorganisms, including *E. coli*. Production of these compounds sometimes requires identifying previously unknown export mechanisms, as suitable transporters have not yet been reported. In such situations, multidrug efflux transporters, with their broad substrate specificity, are promising candidates for exporting metabolites that may otherwise inhibit engineered bacteria.

For example, Wang et al. (2013) demonstrated that coordinated expression of the tripartite AcrA/AcrB/TolC system, which includes a resistance nodulation cell division (RND)-type multidrug transporter, enhanced production of hydrophobic terpenoids such as amorphadiene and kaurene, in engineered *E. coli*. Similarly, overexpressing endogenous *E. coli* transporters—including the MFS-type multidrug efflux transporter MdfA and sugar efflux transporters SetA and SetB—improved lacto-*N*-triose production (Sugita and Koketsu 2022). The MFS transporter MdtH exports the anthocyanin cyanidin 3-O-glucoside (C3G), and increasing its expression raised product titers in metabolically engineered *E. coli* (Wu et al. 2024a). Screening for additional multidrug transporters in *E. coli* led to the discovery of YnfM, which further increased extracellular C3G synthesis by 4.8-fold (Wu et al. 2024b). This case is a representative example showing the validity of screening from a multidrug transporter library.

In addition to their names, multidrug transporters export metabolites that are important to organisms. For instance, flavins, including flavin mononucleotide (FMN) and flavin adenine dinucleotide (FAD), are exported by the multidrug and toxic compound extrusion (MATE)-type transporter YeeO in *E. coli* (McAnulty and Wood 2014). Similarly, the MFS transporter Cs0286 is also involved in the purine nucleotide efflux of *C. stationis*, which is used in the industrial production of XMP and IMP (Kinose et al. 2024). The mutation on this transporter of the industrial strains enhances IMP production by increasing transporter activity. Molecular dynamics simulations could be implied that substituting the 64 th glycine residue in the first transmembrane domain (TM1) of Cs0286 with glutamic acid (G64E) strengthens interaction energies between the α-helices of the inner membrane domains, i.e., between TM1 and TM4 and between TM1 and TM6. This enhanced interaction possibly facilitates transporter movement. Furthermore, the function of MdfA, another MFS transporter in *E. coli*, is similarly improved by strengthening intramolecular salt bridges (Li and Ge 2023). Although some mechanisms remain partially understood, these insights gives an idea of increasing interaction of a particular point of a certain transporter as a strategy for efflux engineering in MFS transporters.

The gain-of-function mechanism of the G64E mutation in Cs0286 may facilitate substrate release from the binding pocket. All-atom molecular dynamics simulations indicated that the substituted 64 th glutamate residue traps the 65 th glutamine residue, which binds to its substrate IMP, facilitating its release (Kinose et al. 2024). Supporting this, a study on the glutamate sodium symporter Glt_Ph_, a member of the excitatory amino acid transporter (EAAT) family, demonstrated that reduced substrate affinity accelerates transport rates (Huysmans et al. 2021). These findings highlight that promoting substrate release can be a viable strategy for improving transporter function. Structural modeling- and simulation-based approaches will support achieving this goal.

## Applications of simulations in transporter engineering

A molecular-level understanding of transport mechanisms is essential for optimizing the function of membrane transporters. In recent years, rapid advancements in molecular dynamics (MD) simulations and structural prediction techniques have facilitated the rational design of transporters, making such approaches increasingly feasible.

As far as we know, studies on MD simulations of efflux transporters in *Corynebacterium* are limited, with only a single reported case concerning a multidrug efflux transporter (Kinose et al. 2024). Since MD simulations require structural information for initial setup, the scarcity of experimentally determined structures for *Corynebacterium* exporters makes the application of modeling techniques, such as homology modeling, particularly challenging. Consequently, the number of MD simulation studies in this area remains low. However, recent advances in structure prediction, particularly the use of AlphaFold2, have enabled the generation of structural models, which may lead to an increase in MD simulation studies in the future.

In contrast, MD simulations have played a crucial role in elucidating the transport mechanisms of multidrug efflux transporters in bacteria beyond *Corynebacterium*, such as *Escherichia coli* and *Mycobacterium tuberculosis*, where crystal structures are available. For instance, in the RND-type transporter AcrB, the functional rotating mechanism (Murakami et al. 2002, 2006) has been demonstrated and supported by MD simulations (Du et al. 2018; Yao et al. 2010). Additionally, MD simulations have provided insights into the conformational changes of AcrB, its stability upon inhibitor binding (Sjuts et al. 2016), and the role of proton binding in drug efflux (Yamane et al. 2013; Matsunaga et al. 2018). Similarly, in the major facilitator superfamily (MFS) transporter MdfA, MD simulations have elucidated the alternating access mechanisms (Law et al. 2008), also known as the rocker-switch mechanism, wherein transitions between the outward-open, inward-open, and occluded states are regulated by protonation (Nagarathinam et al. 2018; Li and Ge 2023). Furthermore, MD simulations of the heterodimeric ABC transporter TM287/288 have demonstrated that ATP and substrate binding induce critical conformational changes, driving the transport cycle and forming states conducive to substrate translocation (Furuta et al. 2016).

The simulation of membrane proteins within realistic membrane environments is crucial for understanding membrane-protein interactions at a molecular level. Advances in computational power, refined force fields, and efficient algorithms have facilitated microsecond scale MD simulations that replicate the biological complexity of membrane systems. These developments have narrowed the gap between computational predictions and experimental observations (Muller et al. 2019). For example, MD simulations employing realistic membrane models demonstrated that peptidoglycans interact with AcrA at the AcrA-TolC interface, stabilizing the AcrAB-TolC complex (Gumbart et al. 2021). In parallel, breakthroughs in structural prediction tools, such as AlphaFold2, have significantly improved the accuracy of transporter modeling. When integrated with MD simulations, these tools enable the optimization of substrate transport pathways and the rational design of transporter variants. For instance, targeted mutations in an MFS transporter involved in IMP transport enhanced specific helical interactions, improving substrate transport efficiency (Kinose et al. 2024). Similarly, the rational design of MmpL4/MmpL5 transporter variants in *Mycobacterium* identified structural elements critical for enhancing substrate transport efficiency and inhibitor sensitivity, providing potential therapeutic applications (Shyam et al. 2024).

Recently, interest in leveraging machine learning (ML) to optimize fermentation-related processes continues to grow. Applications include deep learning approaches to fine-tune microbial metabolic pathways for higher production yields of target compounds (Sabzevari et al. 2022). Additionally, predictive ML models have been used to optimize fermentation conditions, such as modeling growth dynamics in *Candida antarctica* (Sarmah et al. 2022).

The advancement of simulation technologies, structural prediction tools, and ML, or their integration, holds great promise for driving innovations in exporter engineering, advancing fermentation processes, and developing innovative production strategies.

## Conclusions and future perspective

This review highlights the critical role of efflux machinery in microbial production systems. Although identifying specific exporters for each molecule remains challenging, strategies such as exporters for structurally similar molecules (e.g., lysE for non-Lys amino acids) and leveraging efflux carriers with broad substrate specificity suit the design of export systems. Additionally, employing mechanosensitive channels with pore sizes desired for the molecules of interest have possibility of enhancing productivity in certain applications. If applicable, the gain-of-function mutation of MscCG of *C. glutamicum* would help this strategy. When such strategies are inapplicable, initial screening can rely on multidrug efflux transporters. These transporters may enable the removal of undesirable substances and the export of valuable compounds. Computational tools such as MD simulations and advanced structure-prediction platforms offer powerful means to elucidate transporter mechanisms and guide their enhancement. Strengthening transmembrane helix-to-helix interactions, for example, could be one of the simulation-based approaches for improving MFS transporter activity. Further studies are needed to validate this strategy, and it is hoped that progress in this direction will reveal principles for improving transporter function.

Our understanding remains inadequate for accurately predicting transporters and identifying target mutation points for functionality improvement. Future research should focus on elucidating the mechanism of efflux transporters with the aid of computational simulations, structural modeling, ML, and integration of them.
